# Stunted canopy: Marine forests under the thermal effluent of a nuclear power plant

**DOI:** 10.1111/jpy.70113

**Published:** 2025-12-02

**Authors:** Ivan M. Carneiro, Ana Paula A. Veloso, Fábio N. Demarqui, Maria Teresa M. Széchy

**Affiliations:** ^1^ Laboratório Integrado de Ficologia, Departamento de Botânica, Instituto de Biologia, Centro de Ciências da Saúde Universidade Federal do Rio de Janeiro Rio de Janeiro RJ Brazil; ^2^ Departamento de Estatística Universidade Federal de Minas Gerais Belo Horizonte MG Brazil

**Keywords:** chronic disturbance, Fucales, habitat complexity, thallus height, thermal impact

## Abstract

It has been well established that warming beyond certain thresholds can negatively affect the growth of canopy‐forming macroalgae. However, most studies evaluating these effects have been conducted under controlled laboratory conditions. Observational studies investigating the impact of extreme temperatures on growth rates have been rare and typically limited to events such as marine heatwaves or areas affected by thermal pollution. The decline in vertical growth could be detrimental to the development and recovery of macroalgal canopies, significantly impacting habitat complexity. This study examined spatiotemporal variations in thallus height and vertical growth rates of benthic *Sargassum* species near the Brazilian Nuclear Power Station (BNPS). Samples were collected from sites exposed and unexposed to the thermal effluent of the BNPS, across different periods. Throughout the sampling periods, sites exposed to the thermal effluent consistently exhibited lower vertical growth rates than unexposed sites. Reduced thallus height was also observed at impacted sites during the first sampling period, whereas in the second period, this stunted canopy was observed only at the site closest to the thermal effluent outfall. This decline in vertical growth could reduce habitat complexity, potentially altering the structure of shallow rocky macroalgal communities. Even acknowledging the limitations in establishing cause–effect relationships in in situ studies, these results can provide important insights into the potential effects of warming on shallow, rocky‐bottom communities and may offer valuable guidance for managing and monitoring *Sargassum* populations in the face of thermal pollution and global climate change.

**Abbreviations**
BNPS: Brazilian Nuclear Power Station
HRJ: Herbário da Universidade do Estado do Rio de Janeiro
IGB: Ilha Grande Bay
SST: surface seawater temperature

## INTRODUCTION

Habitat complexity of rocky shores is shaped by the geomorphology of the physical substrate and by the characteristics of the dominant sessile organisms, such as canopy‐forming macroalgae. The morphological features of such macroalgae greatly contribute to the three‐dimensional structure of the environment, providing a variety of microhabitats usable by a range of organisms, in some cases with remarkable species‐specific associations (Chen et al., 2020; Chiarore et al., 2019; Marzinelli et al., 2016). In addition to promoting increased diversity and food for grazers, a number of other ecosystem services depends on canopy‐forming macroalgae (Chen et al., 2020, 2021; Coleman & Wernberg, 2017; Eggertsen et al., 2017). Previous studies on macroalgal assemblages have indicated that the loss of canopy‐forming species can alter light availability, sedimentation, scouring, and grazing intensity (Bennett et al., 2015; Bi et al., 2016; Hoey, 2010; Tatsumi et al., 2021). These changes can impact the understory, resulting in the disappearance of some species and the increase in turf‐forming algae (Benedetti‐Cecchi et al., 2001; Bulleri et al., 2002). Consequently, a drastic loss of diversity (Bulleri et al., 2002) and effects on the trophic function of the ecosystem can occur (Chen et al., 2020, 2021).

Along the southeastern Brazilian coast, *Sargassum* species (Phaeophyceae, Fucales) are among the most important canopy‐forming macroalgae on shallow subtidal rocky bottoms (Eston & Bussab, 1990; Széchy & Paula, 2000), providing shelter for fish (Eggertsen et al., 2017; Ornellas & Coutinho, 1998; Teixeira et al., 2009) and invertebrates (Carvalho et al., 2018; Longo et al., 2019; Széchy et al., 2001). The persistence of *Sargassum* beds and consequently their ecological role in a particular area relies, among other processes, on the life‐history traits of the habitat‐former, including its regeneration ability, growth pattern and rate, receptacle differentiation, gamete formation and release, and dispersion of germlings (Ang, 1985a, 2006). Specifically, the elongation of the primary lateral branches of each adult individual, driven by the activity of their apical cell, enables the increase in the canopy height of the population. Having a higher thallus represents an important competitive strategy of *Sargassum*, primarily in terms of space occupancy, light uptake (Critchley et al., 1990; Eston & Bussab, 1990; Olson & Lubchenco, 1990), and gamete production, since the number of fertile receptacles may be positively correlated with thallus height (Sangil et al., 2015). The reestablishment of the canopy height following the period of senescence and shedding of primary lateral branches (Ateweberhan et al., 2005; Széchy et al., 2006) represents a common process in healthy *Sargassum* beds (Ang, 2006), contributing to their persistence and dominance on rocky shores over time. In southeastern Brazil, *Sargassum* species lose their primary lateral branches between June and August, after the reproductive peak, resulting in reduced canopy height. From October to February, with rising temperatures, the optimum growth period occurs, and canopy height is restored through the elongation of primary lateral branches (Széchy et al., 2006). Canopy height can be maintained by the continuous growth of existing branches and by the formation of new ones from the main axes and holdfast, through the regeneration process (Fletcher & Fletcher, 1975; Jensen, 1974; Umezaki, 1986).

The growth rates of *Sargassum* species also depend on abiotic factors, such as nutrient availability (Schaffelke & Klumpp, 1998; Yu et al., 2019), salinity (Li et al., 2019), light intensity, and seawater temperature (Gao & Hua, 1997; Wang et al., 2021; Zou et al., 2018). For instance, Graba‐Landry et al. (2020) assessed the thermal sensitivity of *S. swartzii*, reporting a 17%–49% decrease in the growth of adult thalli when exposed to an increase in seawater temperature of approximately 3.5°C. Moreover, seawater temperatures exceeding 30°C have been identified as detrimental to *Sargassum* species (Ateweberhan et al., 2005; Tsuchiya et al., 2012; Wang et al., 2021), particularly in populations along the Brazilian coast (Chaloub et al., 2025; Gouvêa et al., 2023; Paula, 1994).

Inside Ilha Grande Bay (IGB) located on the southeastern coast of Brazil, the only Brazilian Nuclear Power Station (BNPS) started its commercial activity in 1985 with a single operational unit, and it has been operating with two units since 2000. The seawater used to cool the condensers is released back into the environment as a liquid effluent, constituting a chronic disturbance to the rocky shore communities, especially near the discharge point. This disturbance is characterized by an increase in surface seawater temperature (SST) forming a thermal plume that affects the area around the outfall up to approximately a 3‐km distance (Lucca et al., 2005; Vilanova et al., 2004). The SST near the BNPS's outfall can be up to 8°C higher than that recorded at the control point (Lucca et al., 2005), although there can be considerable variations depending on the season, the oceanographic conditions, and the volume of liquid effluent released into the sea (Mayer‐Pinto et al., 2012). The BNPS's liquid effluent can also change the water flow velocity in front of the outfall and can locally increase the concentration of chlorine, added at the heat exchangers in order to prevent biofouling (Mayer‐Pinto et al., 2012).

Putative negative effects of the BNPS's liquid effluent on *Sargassum* species have been suggested by the decrease in their frequency of occurrence (Széchy et al., 2017) and in their abundance in populations near the outfall (Carneiro et al., 2021, 2024; Teixeira et al., 2009). For instance, Carneiro et al. (2021) reported that after 33 years of activity of the BNPS, extensive *Sargassum* beds disappeared on rocky substrates reached by the thermal plume, while they were still observed at more distant locations inside IGB.

Despite the ecological importance of *Sargassum* canopy for the maintenance of habitat complexity in shallow rocky bottoms, its thallus height, vertical growth rate, and pattern of variation relative to the putative impact of the BNPS's thermal effluent remain largely unknown. The aim of this study was to assess, during a period preceding the disappearance of *Sargassum* beds in the area affected by the thermal plume, whether thallus height and the vertical growth rate of adult *Sargassum* differed between populations from sites exposed to and unexposed to the BNPS's thermal plume.

## MATERIALS AND METHODS

### Study area

This study was conducted in IGB, a 65,258‐hectare coastal bay in southeastern Brazil that includes rocky shores, sandy beaches, and mangroves. A Rapid Marine Assessment conducted in 2003–2004, in depths up to 30 m, revealed the occurrence of over 900 species of macroalgae, invertebrates, and fish (Creed et al., 2007). During this period, *Sargassum* species were dominant in the shallow subtidal rocky bottoms, forming extensive beds in many sites inside IGB (Creed et al., 2007; Széchy & Paula, 2000). In the 1980s, *Sargassum* species were frequent near the BNPS's outfall (Pedrini et al., 1994), and adult individuals measuring more than 30 cm in height were common between October 1981 and August 1983, as derived from herbarium materials originated from the study of Pedrini et al. (1994; Examples: HRJ 7948, HRJ 7949, HRJ 7955, HRJ 8359).

**FIGURE 1:**
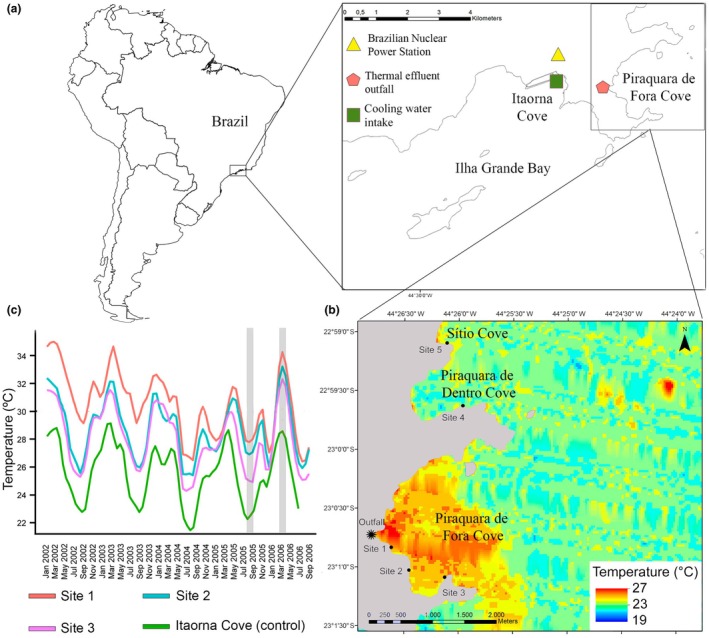
(a) Map of the study area along the southeastern Brazilian coast, showing: Itaorna Cove (where seawater is taken for cooling the BNPS's condensers). (b) Thermal plume dispersion inside Piraquara de Fora Cove evidenced by a Landsat 7 image obtained from USGS EarthExplorer in March 2006. The SST values derived from this image correspond to a single measurement at the time of acquisition; sites 1, 2, and 3 exposed to the thermal plume, inside Piraquara de Fora Cove (where the liquid effluent is discharged). Sites 4 and 5 unexposed to the thermal plume; site 4 inside Piraquara de Dentro Cove and site 5 inside Sítio Cove. (c) Monthly variation of SST at the three sites inside Piraquara de Fora Cove (sites 1, 2, and 3, exposed to the BNPS's thermal plume), and at Itaorna Cove (unexposed to the thermal plume, and considered similar to the control condition) between 2002 and 2006, with the sampling periods highlighted in gray.

In order to dissipate the heat generated by the BNPS's condensers, a pumping system continuously draws seawater from Itaorna Cove and discharges the heated effluent directly into Piraquara de Fora Cove (see Figure 1a). Since there is no urban development or major modifications along the coastline of Piraquara de Fora Cove, the BNPS's liquid effluent was likely the main human‐caused impact in the area during the study period.

The present study included five sites, three located within Piraquara de Fora Cove, and therefore exposed to the thermal plume, and two located outside Piraquara de Fora Cove, unexposed to this disturbance (Figure 1b). The distance from the outfall ranged from 0.34 km at site 1 to 4.68 km at site 5. All sites were sheltered from direct wave action and were comparably exposed to the predominant winds and currents. Similar physicochemical characteristics of surface seawater, particularly salinity and nitrate concentration, were observed between sites inside Piraquara de Fora Cove and adjacent areas outside the influence of the thermal plume (Creed et al., 2007).

### Data gathering and analysis

#### Surface seawater temperature

Surface seawater temperature data were obtained from the Program of Measurement of Seawater Temperature, developed by the BNPS Environmental Monitoring Laboratory, which made biweekly measurements at sites 1, 2, and 3 and at Itaorna Cove from January 2002 to July 2006. Since sites 4 and 5 were not included in the Program of Measurement of Seawater Temperature, SST in the control condition was estimated based on measurements obtained from Itaorna Cove, where seawater is taken up for cooling the BNPS's operational system (Figure 1a,c). Based on summer data from 2003 reported by Creed et al. (2007) for IGB, SST was comparable between sites near Itaorna Cove and those adjacent to sites 4 and 5 of the present study. These findings support the use of Itaorna Cove SST as representative of control conditions in the present study, in contrast to the higher values recorded at sites within Piraquara de Fora Cove.

#### Height and vertical growth rate of *Sargassum*

In previous surveys, the morpho‐species *Sargassum filipendula* and *S. vulgare* were the most common in the study area (Creed et al., 2007; Széchy et al., 2006; Széchy & Paula, 2000). Unfortunately, during fieldwork, assigning each individual to one of these morpho‐species was often difficult or virtually impossible in a consistent way, owing to the widely acknowledged large variability of their blades, one of the main diagnostic characters employed in traditional taxonomic surveys of the genus (Paula, 1988). Moreover, recent molecular analyses have aggregated the two morpho‐species into a single phylogenetic entity (González‐Nieto et al., 2020). Therefore, we considered the *Sargassum* populations from our five sampling sites as belonging to the same phylogenetic species, actually named as *S. natans* (see Wynne, 2022; for a detailed discussion of pertinent taxonomic issues).

The height and vertical growth rate of adult thalli in *Sargassum* populations were evaluated in two periods: August/September 2005 and March/April 2006. The thallus height was estimated based on 40 individuals randomly selected at each site, at 1–2 m depth. The height of the thallus was considered as the length of the longest primary lateral branch, measured from the base of the holdfast to its tip (Espinoza & Rodríguez, 1989; Thomas & Subbaramaiah, 1991). Adult individuals were defined as those with main axes exceeding 1 cm in length (Széchy et al., 2006).

To analyze the vertical growth rate, 20 adult thalli were randomly selected at the same depth (1–2 m) at each site and period. Each thallus was marked using a numbered plastic clip fastened around the main axis. Then, each marked thallus was pruned with sewing scissors, shortening its lateral branches until they were 5 cm in height (measured from the base of the holdfast to the top). This procedure was adopted to standardize the size of the lateral branches, thereby facilitating comparisons among different individuals. Thirty days later, the individuals that remained with the plastic clips (from 10 to 20 individuals) had their longest primary lateral branch measured. The vertical growth rate of each individual was calculated as the difference between the final height and the initial height after pruning (5 cm).

#### Data analysis

The variation of SST at each sampling site was described by line graphs constructed using the Loess smoothing function.

To assess whether there were differences in the *Sargassum* responses between sites exposed or not to the thermal plume, a Control‐Impact sampling design was employed. Comparisons of height and vertical growth rate among sites and periods were performed using linear models with variance adjustments assuming normal distribution, using the R package gamlss (Stasinopoulos et al., 2023), followed by Tukey's post‐hoc multiple comparison tests run with the R package emmeans (Lenth, 2023). This variance‐adjusted model was selected to satisfy the analysis assumption, which was not met even after data transformation. A significance level α = 0.05 was employed in each test.

## RESULTS

### Surface seawater temperature

Throughout the years 2002–2006, SST at Itaorna Cove (control condition) remained consistently below 30°C, ranging between 22°C and 29°C on average. By contrast, SST at the three sites inside Piraquara de Fora Cove, often exceeded 30°C, especially during summer. The SST at site 1 was, in general, higher than at the other two sites, exceeding 34°C on some occasions (Figure 1c).

### Height and growth rate of *Sargassum* adult individuals

**FIGURE 2:**
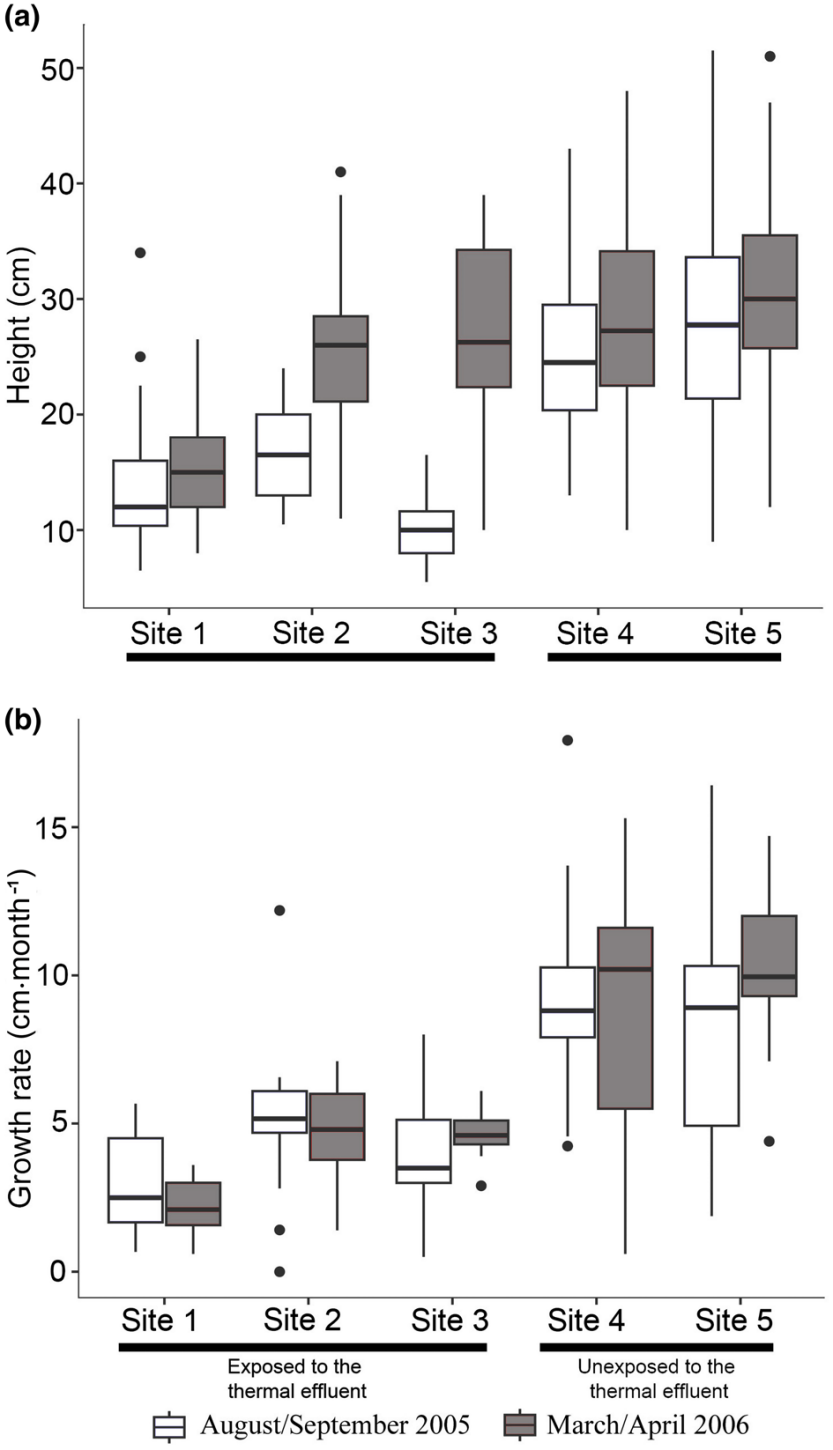
Variation of adult individuals of *Sargassum* at the three sites inside Piraquara de Fora Cove (sites 1, 2, and 3, exposed to the BNPS's thermal plume), and at sites 4 and 5 (not exposed to the thermal plume), in the two study periods: August/September 2005 and March/April 2006. (a) Thallus height (*n* = 40); and (b) growth rate (*n* = 10–20).

The height of *Sargassum* was significantly greater in March/April 2006 than in August/September 2005 at sites 2, 3, and 4, whereas it remained comparable between periods at sites 1 and 5 (Table 1a; Figure 2a). No significant differences in vertical growth rate were detected between the two periods in most sites, only at site 5, where *Sargassum* individuals showed a relatively higher growth rate in March/April 2006 (Table 1b, Figure 2b).

**TABLE 1:** Comparison of adult individuals of *Sargassum* between the two periods (1 = August/September 2005, 2 = March/April 2006) at the five sites around the Brazilian Nuclear Power Station.

Site	Contrast	Estimate	*SE*	*df*	*t* ratio	*p* value
(a) Height						
Site 1	Period 1–2	−2.11	1.1	380	−1.92	0.0553
Site 2	Period 1–2	−9.29	1.34	380	−6.9	**<0.0001**
Site 3	Period 1–2	−17	1.34	380	−12.67	**<0.0001**
Site 4	Period 1–2	−3.69	1.7	380	−2.16	**0.0311**
Site 5	Period 1–2	−2.85	2.04	380	−1.4	0.1626
(b) Growth rate						
Site 1	Period 1–2	0.66	0.52	141	1.26	0.2083
Site 2	Period 1–2	0.46	0.74	141	0.62	0.5379
Site 3	Period 1–2	−0.79	0.47	141	−1.69	0.0927
Site 4	Period 1–2	0.83	1.21	141	0.69	0.4933
Site 5	Period 1–2	−2.15	1.01	141	−2.14	**0.034**

*Note*: (a) Thallus height (*n* = 40); and (b) growth rate (*n* = 10–20). Significant differences are in bold.

Adult individuals of *Sargassum* at sites 4 and 5 showed median height > 20 cm in both periods, whereas such a size was reached only in March/April 2006 at sites 2 and 3 and never at site 1 (Figure 2a).

On average, in August/September 2005, thallus height was higher in populations from sites 4 and 5 than from sites 1, 2, and 3 (Table 2; Figure 2a; Table S1). The smallest thalli were observed at site 3 during this period. In March/April 2006 instead, no significant differences were detected among sites 2, 3, 4, and 5, whereas site 1 was characterized by shorter *Sargassum* individuals compared to all the others (Table 2; Figure 2a).

**TABLE 2:** Comparison of the height of adult individuals of *Sargassum* between paired sites around the Brazilian Nuclear Power Station, in each of two periods of study (*n* = 40).

Contrast	Estimate	*SE*	*df*	*t* ratio	*p* value
(a) Aug/Sep 2005					
Site 1–Site 2	−3.15	1.043	380	−3.021	**0.0225**
Site 1–Site 3	3.38	0.942	380	3.583	**0.0035**
Site 1–Site 4	−11.25	1.331	380	−8.452	**<0.0001**
Site 1–Site 5	−14.46	1.676	380	−8.63	**<0.0001**
Site 2–Site 3	6.53	0.752	380	8.682	**<0.0001**
Site 2–Site 4	−8.1	1.204	380	−6.728	**<0.0001**
Site 2–Site 5	−11.31	1.577	380	−7.175	**<0.0001**
Site 3–Site 4	−14.62	1.118	380	−13.085	**<0.0001**
Site 3–Site 5	−17.84	1.512	380	−11.798	**<0.0001**
Site 4–Site 5	−3.21	1.781	380	−1.804	0.3726
(b) Mar/Apr 2006					
Site 1–Site 2	−10.32	1.389	380	−7.434	**<0.0001**
Site 1–Site 3	−11.51	1.456	380	−7.905	**<0.0001**
Site 1–Site 4	−12.82	1.53	380	−8.381	**<0.0001**
Site 1–Site 5	−15.2	1.596	380	−9.523	**<0.0001**
Site 2–Site 3	−1.19	1.745	380	−0.681	0.9606
Site 2–Site 4	−2.5	1.807	380	−1.383	0.6387
Site 2–Site 5	−4.88	1.863	380	−2.617	0.0694
Site 3–Site 4	−1.31	1.859	380	−0.706	0.9551
Site 3–Site 5	−3.69	1.914	380	−1.927	0.3049
Site 4–Site 5	−2.38	1.971	380	−1.205	0.7484

*Note*: Sites 1, 2, and 3: Exposed to the BNPS's thermal plume; sites 4 and 5: Not exposed to the thermal plume. (a) August/September 2005; (b) March/April 2006. Significant differences are in bold.

In August/September 2005, the mean vertical growth rate of adult individuals of *Sargassum* varied from 2.88 cm · month^−1^ (*SD* ± 1.72; *n* = 14) at site 1, to 9.42 cm · month^−1^ (*SD* ± 3.21; *n* = 18) at site 4. Multiple comparisons indicated that populations from sites 4 and 5 had a higher vertical growth rate than those from sites 1, 2, and 3 during this period (Table 3a).

**TABLE 3:** Comparison of the vertical growth rate of adult individuals of *Sargassum* between paired sites around the Brazilian Nuclear Power Station, in each of two periods of study (*n* = 10–20).

Contrast	Estimate	*SE*	*df*	*t* ratio	*p* value
(a) Aug/Sep 2005					
Site 1–Site 2	−2.2209	0.743	141	−2.989	**0.0269**
Site 1–Site 3	−0.9686	0.601	141	−1.611	0.4932
Site 1–Site 4	−6.5391	0.86	141	−7.605	**<0.0001**
Site 1–Site 5	−5.287	0.957	141	−5.523	**<0.0001**
Site 2–Site 3	1.25235	0.72	141	1.74	0.413
Site 2–Site 4	−4.3182	0.946	141	−4.563	**0.0001**
Site 2–Site 5	−3.0661	1.036	141	−2.96	**0.0292**
Site 3–Site 4	−5.5706	0.84	141	−6.633	**<0.0001**
Site 3–Site 5	−4.3184	0.939	141	−4.597	**0.0001**
Site 4–Site 5	1.25213	1.122	141	1.115	0.7981
(b) Mar/Apr 2006					
Site 1–Site 2	−2.4229	0.525	141	−4.616	**0.0001**
Site 1–Site 3	−2.4217	0.362	141	−6.697	**<0.0001**
Site 1–Site 4	−6.3695	0.999	141	−6.375	**<0.0001**
Site 1–Site 5	−8.1022	0.608	141	−13.33	**<0.0001**
Site 2–Site 3	0.00119	0.504	141	0.002	1
Site 2–Site 4	−3.9466	1.059	141	−3.726	**0.0026**
Site 2–Site 5	−5.6794	0.702	141	−8.089	**<0.0001**
Site 3–Site 4	−3.9478	0.988	141	−3.994	**0.001**
Site 3–Site 5	−5.6806	0.59	141	−9.627	**<0.0001**
Site 4–Site 5	−1.7328	1.103	141	−1.572	0.5181

*Note*: Sites 1, 2 and 3: Exposed to the BNPS's thermal plume; sites 4 and 5: Not exposed to the thermal plume. (a) August/September 2005; (b) March/April 2006. Significant differences are in bold.

In March/April 2006, the mean vertical growth rate of adult individuals of *Sargassum* varied from 0.22 cm · month^−1^ (*SD* ± 0.91; *n* = 10) in site 1 to 10.32 cm · month^−1^ (*SD* ± 2.36; *n* = 18) at site 5. In this period, significant differences were detected between the population from site 1 and all the others. The populations from sites 4 and 5 had higher vertical growth rates than those from the other three sites (Table 3b).

## DISCUSSION

The negative effects of elevated temperatures exceeding the physiological limits of macroalgae have been demonstrated in multiple experimental studies across different species and regions (Hurd et al., 2014; Ji & Gao, 2021; Koch et al., 2013). Although thallus growth has been a commonly assessed variable in short‐term manipulative experiments, the effect of increased temperature on *Sargassum* populations exposed to an in situ chronic disturbance from a thermal effluent had not yet been evaluated. The reduced vertical growth rate observed at sites exposed to the thermal effluent in different periods suggests that this chronic disturbance negatively impacts the canopy development of these populations. At the site closest to the effluent outfall, the reduced growth rate was accompanied by smaller thallus height regardless of the study period, indicating that canopy height did not recover and remained persistently stunted. Manipulative studies have shown that temperatures above 30°C, commonly observed during summer at sites exposed to the chronic disturbance from the thermal effluent, generally exceed the physiological limits of *Sargassum* populations from warm‐temperate regions (Gouvêa et al., 2023; Graba‐Landry et al., 2020). Experiments conducted with *Sargassum* populations from IGB and surrounding areas exposed to these high temperatures revealed negative effects on carbon and nitrogen metabolism, as well as reductions in photosynthetic efficiency and primary productivity (Chaloub et al., 2025; Urrea‐Victoria et al., 2020).

The lower growth rates also observed at some sites inside Piraquara de Fora Cove in August/September 2005, when SST did not exceed 30°C, offer insights regarding the possible long‐term effects of seawater warming on *Sargassum* canopies. Firstly, long exposure to such high temperatures can have a prolonged effect on macroalgal thalli by causing denaturation or inactivation of proteins and enzymes leading to permanent cytological damage with consequences for photosynthetic efficiency and primary productivity (Eggert, 2012; Shen et al., 2025; Simonson et al., 2015). Such impairments can persist even when environmental conditions later return to favorable levels, causing declines up to the extinction of the population (Montie & Thomsen, 2023). Secondly, but not mutually exclusively, the chronic disturbance related to the continuous elevated SST inside Piraquara de Fora Cove may have reduced the genetic diversity within local *Sargassum* populations, favoring haplotypes linked to lower growth rates. Such a reduction in genetic diversity was documented in subtidal macroalgal forests in Australia after an extreme marine heatwave in 2011 (Gurgel et al., 2020). Based on our study, it is not possible to discern to what extent the growth rates estimated for the IGB's populations of *Sargassum* are attributable to genetic peculiarities of the target species and populations beyond the effects of the environmental conditions.

Finally, other explanatory models for the reduction of the growth rate of *Sargassum* could involve the interaction between high temperature and organisms associated with the algal thalli. Seawater temperature can affect the structure and composition of microbial communities inhabiting the thallus (Mensch et al., 2020), leading to changes in their capacities to uptake nutrients and vitamins (Duarte et al., 2018; Schaffelke, 1999). Moreover, warming can increase grazing pressure on macroalgae (Bernal‐Ibáñez et al., 2022; Duarte et al., 2018), causing thallus damage. Higher density of *Chelonia mydas*, an important grazer in the region, was recorded closer to the outlet in comparison with control sites (Pereira et al., 2021). Specifically designed experimental studies are needed to test these hypotheses in IGB and to improve our understanding of the effects of seawater warming on *Sargassum* growth.

Increased SST can also affect other processes linked to the developmental stages of the species, specifically the senescence process (Ang Jr, 1985b; Ateweberhan et al., 2005), which can explain the considerable variation in the thallus height of adult individuals among sites. High temperature can cause damage to the tissues of canopy‐forming algae, making the thallus more susceptible to breakage (Simonson et al., 2015). Warming may have intensified the process of lateral branch loss in the sites under the effect of the thermal plume, especially during the senescence period, leading to lower height during August/September 2005. However, the lower thallus height at site 3 compared to site 1 indicates that other local disturbances may be interacting with temperature, affecting thallus height.

The reduced vertical growth rate of *Sargassum*, observed in this study for sites inside Piraquara de Fora Cove, can be considered one of the processes related to the long‐term reduction in the frequency and cover of *Sargassum* in populations exposed to the BNPS's thermal plume (Carneiro et al., 2024; Széchy et al., 2017). *Sargassum* growth rate may be a more suitable descriptor than frequency and cover for detecting environmental disturbances in the short term. Assessing spatial and temporal variations in the growth rate of *Sargassum* can improve the prediction of changes in structural variables of populations and communities, such as population density and relative abundance of species, which are generally applied in medium and long‐term monitoring studies. In the context of warming associated with thermal pollution or climate change, it is essential to prioritize ecological and physiological indicators with rapid response times to detect ecosystem changes more effectively.

From a methodological point of view, we acknowledge that the adopted Control‐Impact approach, required because of the lack of baseline data on *Sargassum* life‐history traits before the start of the BNPS's operations, calls for caution when interpreting our results (Underwood, 1994). Nevertheless, herbarium materials indicated that adult *Sargassum* individuals exceeding 30 cm in height were common at site 1 and nearby areas before the establishment of the BNPS, supporting the interpretation that the thermal plume may have impacted *Sargassum* vegetative development. The lack of sites unaffected by the thermal plume inside Piraquara de Fora Cove also prevented us from fully disentangling the potential effects of other local processes acting on different coves. However, the absence of significant differences in *Sargassum* height and growth rate between the two control sites, also located in different coves, allowed us to consider potential local drivers of variability, such as sedimentation and water movement (Carneiro et al., 2020), as likely of minor influence compared to the effects of the BNPS's thermal effluent.

This study highlights the detrimental impact of seawater warming on *Sargassum* populations from IGB and strengthens the role of this species as an indicator of thermal pollution and climate‐related alterations in coastal environments of Brazil and elsewhere. Similar evidences were provided by observational (Carneiro et al., 2021, 2024; Gorman et al., 2020; Széchy et al., 2017), manipulative (Gouvêa et al., 2023; Urrea‐Victoria et al., 2020), and species‐distribution modeling studies (Carneiro et al., 2023; Faga & Gurgel, 2024). The reduced vertical growth and canopy height observed in sites exposed to the thermal effluent suggest that stunted *Sargassum* canopies could occur elsewhere if temperatures exceed 30°C under future climate change scenarios. Such reductions could diminish habitat complexity and disrupt the structure and dynamics of rocky shore communities, affecting turtles, fish, and invertebrates that rely on this habitat (Eggertsen et al., 2017; Reisser et al., 2013; Széchy et al., 2001).

## AUTHOR CONTRIBUTIONS

**Ivan M. Carneiro:** Conceptualization (equal); data curation (lead); formal analysis (lead); methodology (equal); supervision (equal); validation (equal); visualization (equal); writing – original draft (lead); writing – review and editing (lead). **Ana Paula A. Veloso:** Data curation (equal); investigation (equal); methodology (equal); writing – original draft (supporting). **Fábio N. Demarqui:** Data curation (equal); formal analysis (equal); methodology (equal); writing – original draft (equal); writing – review and editing (equal). **Maria Teresa M. de Széchy:** Conceptualization (equal); investigation (lead); methodology (equal); project administration (lead); resources (lead); supervision (lead); writing – original draft (equal); writing – review and editing (equal).

## Supporting information


**Table S1.** Global tests of linear models with variance adjustments comparing mean values of (a) height and (b) growth rate.
